# Increased Prostaglandin E2 in Brainstem Respiratory Centers Is Associated With Inhibition of Breathing Movements in Fetal Sheep Exposed to Progressive Systemic Inflammation

**DOI:** 10.3389/fphys.2022.841229

**Published:** 2022-03-03

**Authors:** Vanesa Stojanovska, John Atta, Sharmony B. Kelly, Valerie A. Zahra, Eva Matthews-Staindl, Ilias Nitsos, Alison Moxham, Yen Pham, Stuart B. Hooper, Eric Herlenius, Robert Galinsky, Graeme R. Polglase

**Affiliations:** ^1^The Ritchie Center, Hudson Institute of Medical Research, Melbourne, VIC, Australia; ^2^Department of Obstetrics and Gynaecology, Monash University, Melbourne, VIC, Australia; ^3^Department of Women’s and Children’s Health, Karolinska Institute, Karolinska University Hospital, Stockholm, Sweden; ^4^Astrid Lindgren Childrens Hospital, Karolinska University Hospital, Stockholm, Sweden

**Keywords:** brainstem, respiratory centers, fetal breathing movements, inflammation, PGE_2_

## Abstract

**Background:**

Preterm newborns commonly experience apnoeas after birth and require respiratory stimulants and support. Antenatal inflammation is a common antecedent of preterm birth and inflammatory mediators, particularly prostaglandin E2 (PGE_2_), are associated with inhibition of vital brainstem respiratory centers. In this study, we tested the hypothesis that exposure to antenatal inflammation inhibits fetal breathing movements (FBMs) and increases inflammation and PGE_2_ levels in brainstem respiratory centers, cerebrospinal fluid (CSF) and blood plasma.

**Methods:**

Chronically instrumented late preterm fetal sheep at 0.85 of gestation were randomly assigned to receive repeated intravenous saline (*n* = 8) or lipopolysaccharide (LPS) infusions (experimental day 1 = 300 ng, day 2 = 600 ng, day 3 = 1200 ng, *n* = 8). Fetal breathing movements were recorded throughout the experimental period. Sheep were euthanized 4 days after starting infusions for assessment of brainstem respiratory center histology.

**Results:**

LPS infusions increased circulating and cerebrospinal fluid PGE_2_ levels, decreased arterial oxygen saturation, increased the partial pressure of carbon dioxide and lactate concentration, and decreased pH (*p* < 0.05 for all) compared to controls. LPS infusions caused transient reductions in the % of time fetuses spent breathing and the proportion of vigorous fetal breathing movements (*P* < 0.05 vs. control). LPS-exposure increased PGE_2_ expression in the RTN/pFRG (*P* < 0.05 vs. control) but not the pBÖTC (*P* < 0.07 vs. control) of the brainstem. No significant changes in gene expression were observed for PGE_2_ enzymes or caspase 3. LPS-exposure reduced the numbers of GFAP-immunoreactive astrocytes in the RTN/pFRG, NTS and XII of the brainstem (*P* < 0.05 vs. control for all) and increased microglial activation in the RTN/pFRG, preBÖTC, NTS, and XII brainstem respiratory centers (*P* < 0.05 vs. control for all).

**Conclusion:**

Chronic LPS-exposure in late preterm fetal sheep increased PGE_2_ levels within the brainstem, CSF and plasma, and was associated with inhibition of FBMs, astrocyte loss and microglial activation within the brainstem respiratory centers. Further studies are needed to determine whether the inflammation-induced increase in PGE_2_ levels plays a key role in depressing respiratory drive in the perinatal period.

## Introduction

The ability to breathe is critical for survival. However, for many babies that are born preterm, the ability to breathe is severely compromised. Indeed, up to 92% of very preterm babies (<32 weeks’ gestation) and 91% of moderate to late preterm babies (32–36 weeks’ gestation) require mechanical respiratory support to survive (Chow et al., 2017). While life-saving, there is strong evidence that mechanical respiratory support causes systemic and cerebral inflammation, resulting in white matter injury and an increased risk of neurodevelopmental impairments, such as cerebral palsy (Polglase et al., 2012a,b, 2014; Galinsky et al., 2018b). Approximately 40–70% of very preterm babies are exposed to infection and/or inflammation *in utero* (Tita and Andrews, 2010). Intrauterine inflammation increases the risk of preterm birth, increases the requirement for respiratory support and further increases the risk and severity of preterm brain injury (Zhao et al., 2011; Polglase et al., 2012b; Tapia et al., 2016; Stojanovska et al., 2018a,b). Thus, improving our understanding of the mechanisms that underpin inflammation-induced impairments in breathing could lead to improved interventions that reduce the requirement for respiratory support after birth, and in turn reduce the incidence and severity of adverse neurodevelopmental outcomes.

Adverse *in utero* conditions are known to dysregulate fetal and postnatal breathing (Koos and Rajaee, 2014; Baburamani et al., 2021). While fetal breathing movements (FBMs) play no role in gas exchange, they are critical for lung growth, the development of respiratory-related muscles and for training respiratory control centers within the brainstem for continuous breathing after birth (LoMauro and Aliverti, 2016). FBMs (and breathing in general) are generated and regulated by a large network of neurons or “respiratory centers” within the brainstem (Smith et al., 2009). These respiratory centers are critical for generating respiratory rhythm, modulating inspiratory/expiratory timing and effort, processing and adapting to central and peripheral chemosensory information (carbon dioxide, pH, and oxygen levels) and controlling muscles important for maintaining airway patency and breathing biomechanics (Smith et al., 2009; Smith et al., 2013). Studies in fetal sheep and neonatal mice have demonstrated that inflammatory mediators such as prostaglandins [namely prostaglandin E2 (PGE_2_)] and cytokines (e.g., IL-1) are associated with inhibition of fetal and neonatal respiratory function (Wlodek et al., 1998; Hofstetter et al., 2007; Siljehav et al., 2014; Siljehav et al., 2015). Intravenous infusion of PGE_2_ to fetal sheep depresses FBMs, whereas prostaglandin synthesis inhibitors stimulate breathing (Wlodek et al., 1998). High PGE_2_ levels are associated with hypoventilation, a reduction in respiratory frequency, and apnoeas in neonatal mice (Siljehav et al., 2012). Preterm human infants have higher cerebrospinal fluid (CSF) PGE_2_ levels compared to their term counterparts. Indeed, CSF PGE_2_ levels were highest in preterm infants exposed to perinatal infection/inflammation (Siljehav et al., 2015), and were positively associated with apnoeic and hypoxic events (Siljehav et al., 2015).

Although the link between elevated systemic and CSF PGE_2_ levels and breathing inhibition has been demonstrated in the neonate, whether this relationship exits in the fetus, and is associated with accumulation of PGE_2_ and inflammation in the brainstem respiratory centers remains unknown. Therefore, the aims of this study were to determine whether: (1) antenatal inflammation inhibits FBMs, and (2) if changes in FBMs are associated with inflammation and high PGE_2_ levels in the brainstem respiratory centers, CSF and plasma. We hypothesized that increased brainstem inflammation and PGE_2_ levels during antenatal inflammation are associated with a reduction in FBMs.

## Materials and Methods

### Ethics Statement

All procedures were approved by the Hudson Institute of Medical Research Animal Ethics committee and were conducted in accordance with the National Health and Medical Research Council Code of Practice for the Care and Use of Animals for Scientific Purposes (Eighth Edition).

### Fetal Instrumentation

Sixteen pregnant Border-Leicester ewes bearing singleton or twin fetuses underwent aseptic surgery at either 124 or 125 days of gestation (term = 147 days). Food but not water was withdrawn approximately 18 h before surgery. Anesthesia was induced by i.v injection of sodium thiopentone (20 mL) and maintained using 2–3% isoflurane in oxygen (Bomac Animal Health, Hornsby, NSW, Australia). Ewes received prophylactic antibiotics (ampicillin, 1 g i.v.; Austrapen, Lennon Healthcare, St. Leonards, NSW, Australia, and engemycin, 500 mg i.v.; Schering-Plough, Upper Hutt, New Zealand) immediately before surgery. Isoflurane levels, heart rate, and respiratory rate were continuously monitored throughout surgery by trained anesthetic staff.

A midline maternal laparotomy was performed, the fetus was exposed, and polyvinyl catheters were inserted into the trachea (for measurement of fetal breathing *via* tracheal pressure), right brachiocephalic artery (for serial arterial blood gas measurements and plasma collection), brachial vein [for administration of lipopolysaccharide (LPS) or vehicle] and amniotic cavity (to correct fetal tracheal pressure for maternal movement). In the case of a twin pregnancy, only one twin was instrumented. The fetus was returned to the uterus in its original orientation and all fetal leads were exteriorized through the maternal flank. A catheter was inserted into the maternal jugular vein for administration of post-operative antibiotics and euthanasia at the end of the experimental period. At the completion of surgery, ewes received fentanyl for 3 days *via* a transdermal patch placed on the left hind leg (75 μg/h; Janssen Cilag, North Ryde, NSW, Australia).

Ewes were housed together in separate metabolic crates in a temperature-controlled room (20 ± 2°C and relative humidity of 50 ± 10%) with a 12-h light-dark cycle with *ad libitum* access to food and water. Four to five days of postoperative recovery was allowed before experiments commenced. Ewes and fetuses received daily i.v. infusions of ampicillin (800 mg, maternal i.v. and 200 mg, fetal i.v.) and engemycin (500 mg, maternal i.v.) for three consecutive days after surgery. All ewes were deemed healthy throughout the experimental timeline, as indicated by laboratory monitoring records. Fetal catheters were maintained patent with a continuous infusion of heparinised saline (25 IU/mL) at a rate of (0.2 mL/h).

### Experimental Recordings

Continuous recordings of tracheal pressures (as a measure of FBMs) and amniotic pressure began 24 h prior to the first saline/LPS infusion (at 129 days of gestation) and continued until the end of the experiment (at 134 days of gestation) using LabChart pro software (Version 8, ADInstruments, Castle Hill, NSW, Australia). Amniotic and tracheal pressures were measured using pressure transducers (cat# ADInstriuments, cat# MLT0699) connected to a quad bridge amp and powerlab (ADInstriuments, cat# FE224 and PL3508). The tracheal and amniotic pressure signals were collected at 1 kHz using a mains filter and stored offline for analyses using LabChart pro (version 8, ADInstruments).

### Experimental Protocol

Experiments started at 129 days of gestation. Fetuses were randomly allocated to receive either saline (control, *n* = 8) or LPS infusions (*Escherichia coli*, 055:B5, MilliporeSigma, Burlington, MO, United States, n = 8). Fetuses received 300, 600, and 1200 ng infusions of LPS diluted in 2 mL of saline i.v. (infusion rate: 1 mL/min) on experimental days 1, 2, and 3, respectively, as previously described (Kelly et al., 2021). This experimental model is relevant to the acute inflammatory exacerbations observed during perinatal infection/inflammation, which is associated with adverse neurodevelopmental outcomes (Grether and Nelson, 1997; Yanowitz et al., 2002). The incremental LPS infusions reduce tolerance to subsequent LPS infusions, when compared to using repeated LPS infusions of the same dose (Rees et al., 2010; Kelly et al., 2021), thereby allowing us to more closely mimic the repeated inflammatory events observed in infants with an increased risk of developing neural injury (Kuban et al., 2014). Controls received an equivalent volume of saline at the same infusion rate. Fetal preductal arterial blood samples were collected every morning (0900 h) starting from 30 min before the start of the experiment until the day of post-mortem for pH, blood gases, and glucose and lactate concentrations (ABL 90 Flex Plus analyser, Radiometer, Brønshøj, Denmark).

Four days after the start of infusions, sheep were euthanized by intravenous injection (8 g) of pentobarbitone sodium (Lethabarb, Virbac, NSW, Australia).

### Fetal PGE_2_ Measurements

Additional blood samples were collected immediately before LPS or saline infusions (baseline), and +2 and +6 h after LPS/saline infusions and CSF was collected at post-mortem for measurement of PGE_2_ levels using a commercially available bovine monoclonal PGE_2_ ELISA kit (cat# 514010, Cayman Chemicals, Ann Arbor, MI, United States) according to the manufacturer’s instructions. In brief, 50 μl samples were added to the 96 well plates in triplicate, tracer and monoclonal antibodies were added and incubated overnight at 4°C. The plates were washed to remove any unbound reagents. Ellman’s reagent was added to each well and incubated at room temperature for 1.5 h. The plates were read at 410 nm on a plate reader (SpectraMax i3, Molecular Devices, San Jose, CA, United States). Internal quality controls were included in each assay and PGE_2_ levels were within the detection limit in all samples. The intra-assay and inter-assay coefficient of variability (%CV) was set to <20% %CV. Assay range: 7.8–1,000 pg/ml and sensitivity (defined as 80% B/B_0_): 15 pg/ml.

### Fetal Breathing Movements

Fetal breathing movements were identified as phasic reductions in tracheal pressure greater than 1.5 mmHg, as previously described (Polglase et al., 2004). Data were collected at the same time of day to avoid diurnal changes in FBMs (Houghton et al., 1993). FBM data were analyzed on experimental days 1, 2, and 3 before LPS/saline infusion (baseline) and at 0 h (the hour of saline/LPS infusion), +1 h, +2 h, +3 h, +4 h, +5 h, +6 h, +12 h after saline/LPS infusions. The total time spent breathing was expressed as the % of time FBMs occurred per hour. FBMs were further analyzed to assess: (i) the average duration of FBMs (s) per hour; (ii) the frequency of FBMs (average number of breaths/second during an episode of FBMs over each 1 h epoch); (iii) the amplitude of FBMs (average depth of breaths during each 1 h epoch); and (iv) the % of vigorous FBMs. Vigorous FBMs were defined by a reduction in tracheal pressure ≥ 5 mmHg).

### Cerebrospinal Fluid and Brainstem Collection and Processing

Cerebrospinal fluid (CSF) was collected from the fourth ventricle lining the brainstem by introducing a 21G needle through the cisterna magna and was immediately frozen in liquid nitrogen, samples were successfully collected from 6/8 control and 6/8 LPS-exposed subjects. The brainstem was removed from the cerebellum and cerebrum at the level of peduncles and thalamus and sectioned along the midline. The left brainstem was frozen in liquid nitrogen in 7/8 control and 7/8 LPS-exposed subjects. The right brainstem was immersion fixed in 10% neutral buffered formalin for 6 days prior to paraffin processing and embedding. Serial 8 μm sections of the brainstem were cut and brainstem respiratory centers were visually identified using the Michigan State Sheep Brain atlas^1^ the rat brain atlas (Paxinos, 1986) and the study by Nitsos and Walker (1999). The putative retrotrapezoid/parafacial respiratory group (RTN/pFRG) area was identified ventral to the facial nucleus in sections of the medulla oblongata. This region is involved with expiration and central chemosensitivity (CO_2_ and pH). The nucleus tractus solitarius (NTS), hypoglossal nucleus (XII), putative preBötzinger complex (preBÖTC), and raphe obscurus nucleus were all imaged from the same sections taken above the level of the central canal. Landmarks for identifying the preBÖTC included the NTS, XII, nucleus ambiguous, and the inferior olivary nucleus. The NTS is responsible for peripheral chemosensitivity (O_2_, CO_2_, pH), XII is responsible for upper airway patency, the raphe nucleus is responsible for central chemosensitivity, and the preBÖTC is responsible for rhythmic generation of inspiratory drive.

### Immunohistochemistry

Brainstem sections were incubated at 60°C for 30 min, dewaxed in xylene and ethanol solutions (100%, 70%), rinsed in water and in 1x phosphate buffered solution (1x PBS). For PGE_2_ immunolabelling, ready-to-use Proteinase K (Sigma-Aldrich, North Ryde, NSW, Australia) antigen retrieval was performed at room temperature for 12 min. Sections were washed with 1x PBS 3x for 5 min and were blocked using 10% normal goat serum (NGS) diluted in 1x PBS for 30 min. For immunolabelling of glial fibrillary acidic protein (GFAP; astrocytes) and ionized calcium binding protein 1 (IBA-1; microglia), heat-mediated antigen retrieval was performed using citrate buffer, pH 6, 3x for 5 min. For GFAP and IBA-1 labeling, sections were incubated in an antibody blocking solution consisting of 10% NGS and 0.1% Triton X-100 in 1x PBS for 30 min at room temperature. Following the 30 min incubation, the antibody blocking solution was decanted and all sections were washed 1x for 5 min with 1x PBS. Primary antibodies: anti-rabbit PGE_2_ (1:200; Abcam, Melbourne, Australia), anti-mouse GFAP (1:500; Abcam, Australia), and anti-rabbit IBA-1 (1:500; WAKO Chemicals, Osaka, Japan) were incubated overnight at 4°C. Sections were washed in 1x PBS 3x for 10 min, then incubated with a respective secondary antibody: anti-rabbit Alexa Fluor 488 for PGE_2_ (1:150) and IBA-1 (1:200), and anti-mouse Alexa Fluor 594 for GFAP (1:200) all sourced from Jackson Immuno Research, United States. Sections were washed in 1x PBS 3x for 10 min then incubated with the nuclear stain HOECHST (1:1000 diluted in 1x PBS; Invitrogen, Waltham, Massachusetts, United States) for 5 min, then washed 1x for 5 min in 1x PBS. Sections were cover-slipped using DAKO anti-fade fluorescent mounting medium (Agilent Technologies, Santa Clara, CA, United States) and sealed using clear nail varnish and allowed to dry.

### RT-PCR–Gene Expression of PGHS-2, PGES, and CASP3

RT-PCR was performed on 150 mg tissue from the medulla oblongata. Tissue was homogenized and total RNA was isolated (RNeasy Midi Kit, Qiagen, Germantown, MD, United States) as per manufacturer’s instructions. DNA digestion was performed using the RNase-free DNase kit (Qiagen, Australia). RNA purity and concentration were measured using a spectrophotometer (Nandodrop, Thermofisher Scientific, Waltham, MA, United States), and gel electrophoreses was performed to validate the quality of extracted RNA and to determine any DNA contamination. RNA was reverse-transcribed into cDNA (SuperScript III reverse transcriptase, Invitrogen). Genes of interest were measured by RT-PCR using the Applied Biosystems QuantStudio 6 Flex Real-Time PCR system (Thermo Fisher Scientific, Melbourne, Australia). Relative mRNA expression of cyclooxygenase-2 (COX-2; gene name *PGHS-2*), prostaglandin E synthase (*PGES*), and caspase 3 (*CASP3*) were measured. *COX-2* and *PGES* was chosen as they catalyze the production of PGE2. *CASP3* was chosen to assess cell death. Gene expression was normalized to 18S mRNA for each sample using the cycle threshold (ΔCT) method of analysis and expressed as a fold change relative to the saline group.

### Imaging and Quantitative Analysis

All fluorescent images were taken at 40x magnifications using an Olympus BX50 microscope and Cell Sense imaging software (Olympus, Tokyo, Japan). For each subject, a total of four non-overlapping images were taken per respiratory center. No staining was observed in negative controls where the primary antibodies were omitted. Slides and images were coded and the observer (VS) was blinded to the treatment. The % area coverage of PGE_2_ labeling was assessed in four non-overlapping fields per brainstem region using a set intensity threshold (FIJI; ImageJ, NIH Image, MD, United States). Fibrous and protoplasmic GFAP + astrocytes were included in the analysis. Microglia were categorized and counted with respect to their morphological phenotype. This analysis included: (1) resting ramified microglia (small cell body with fine processes), (2) hyper-ramified microglia (enlarged cell body with extensive fine processes), (3) reactive microglia (enlarged cell body with thickened and retracted processes), and (4) ameboid/phagocytic microglia (ameboid in shape and devoid of processes), as previously described (Morrison et al., 2017; Galinsky et al., 2020b; Nott et al., 2020). All counts were normalized and expressed as mean total number of cells/mm^2^ area for each brainstem region.

### Statistical Analysis

Physiological data (blood gases, FBMs, and plasma PGE_2_ levels) were analyzed using a 2-Way ANOVA with repeated measures and a Sidak’s multiple comparisons test, with treatment and time as independent factors. Normality tests were performed prior to undertaking statistical analyses. Immunohistochemical, CSF ELISA, and PCR data were analyzed using an unpaired Student’s *t*-test. All analyses were performed using GraphPad Prism v8 (GraphPad Software, San Diego, CA, United States). Data are presented as mean ± SEM, unless stated otherwise. Values of *p* < 0.05 were considered statistically significant.

## Results

### Baseline Period

Prior to LPS exposure there were no differences in fetal blood biochemistry and fetal breathing movements between groups.

### Fetal Characteristics

There were no significant differences in the ratio of males to females, singletons to twins, body weight, brain weight, or lung weight. Spleen weight was increased in LPS-exposed fetuses compared to controls (*P* < 0.05, Table 1).

**TABLE 1:** Fetal characteristics. *P < 0.05 vs control (SAL).

	SAL	LPS
Number (N=)	8	8
Singletons, twins (N=)	5, 3	5, 3
Females, males (N=)	3, 5	1, 7
Body weight (kg)	4.1 ± 0.2	4.4 ± 0.2
Brain weight (g)	51.0 ± 1.0	50.0 ± 1.2
Lung weight (g)	168 ± 7.5	159 ± 17
Spleen weight (g)	8 ± 1.0	10.1 ± 0.9*

### Fetal Blood Gases

**FIGURE 1:**
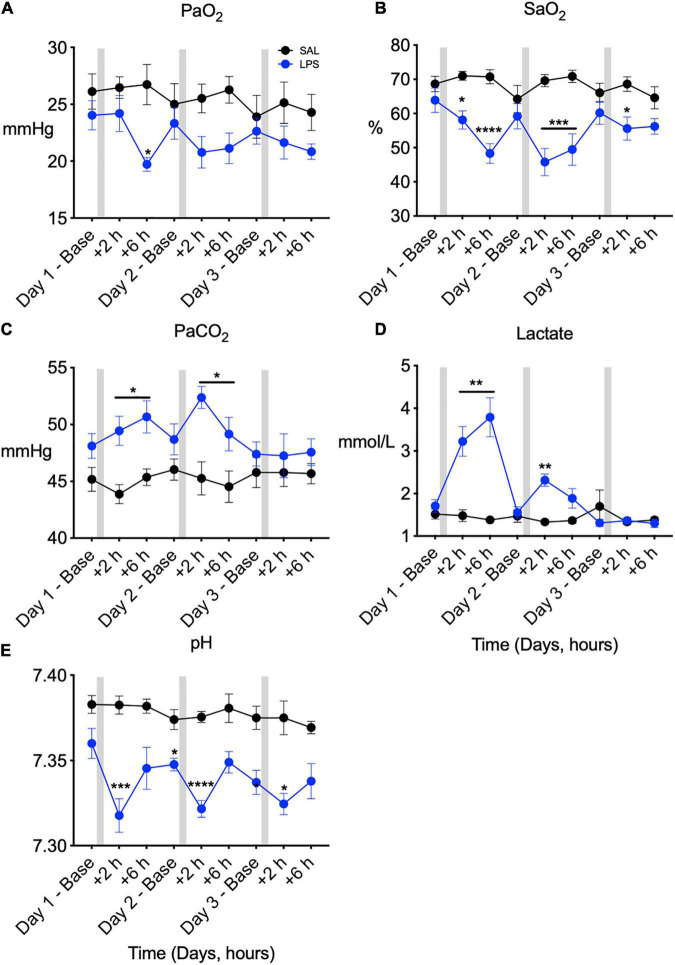
Fetal arterial blood gases measured across 3 days at baseline (0 h), +2 h, and +6 h following saline [SAL (control)]/LPS infusions. Partial pressure of oxygen (PaO_2_, **A**), oxygen saturation (SaO_2_, **B**), partial pressure of carbon dioxide (PaCO_2_, **C**), lactate **(D)**, and pH **(E)** in SAL (control = black) and LPS-exposed (blue) fetuses. Gray bars indicate SAL or LPS infusion on each day after baseline sampling. Data are means ± SEM, *P < 0.05 vs. control, **P < 0.01 vs. control, ***P < 0.001 vs. control, ****P < 0.0001 vs. control.

In LPS exposed fetuses, PaO_2_ was lower compared to control at +6 h on day 1 after LPS infusion (*P*treat < 0.05, *P*treat × time = 0.11, Figure 1A). SaO_2_ was lower in LPS-exposed fetuses compared to controls on days 1, 2, and 3 after LPS infusions (*P*treat < 0.01, *P*treat × time < 0.00, days 1 and 2 + 2 and + 6 h, and day 3 + 2 h, Figure 1B). PaCO_2_ was higher in LPS-exposed fetuses compared to controls on days 1 and 2 after LPS infusions (*P*treat = 0.02, *P*treat × time = 0.01 days 1 and 2, +2 and +6 h, Figure 1C). Arterial lactate concentration was higher in LPS-exposed fetuses compared to control on days 1 and 2 after LPS infusions (*P*treat = 0.01, *P*treat × time < 0.00, day 1 + 2 h and + 6 h and day 2 + 2 h, Figure 1D). In LPS-exposed fetuses, pH was lower than control from day 1 to day 3 (*P*treat < 0.00, *P*treat × time = 0.02, day 1 + 2 h, day 2 baseline and + 2 h, day 3 baseline and + 2 h, Figure 1E).

### Fetal Breathing Movements

**FIGURE 2:**
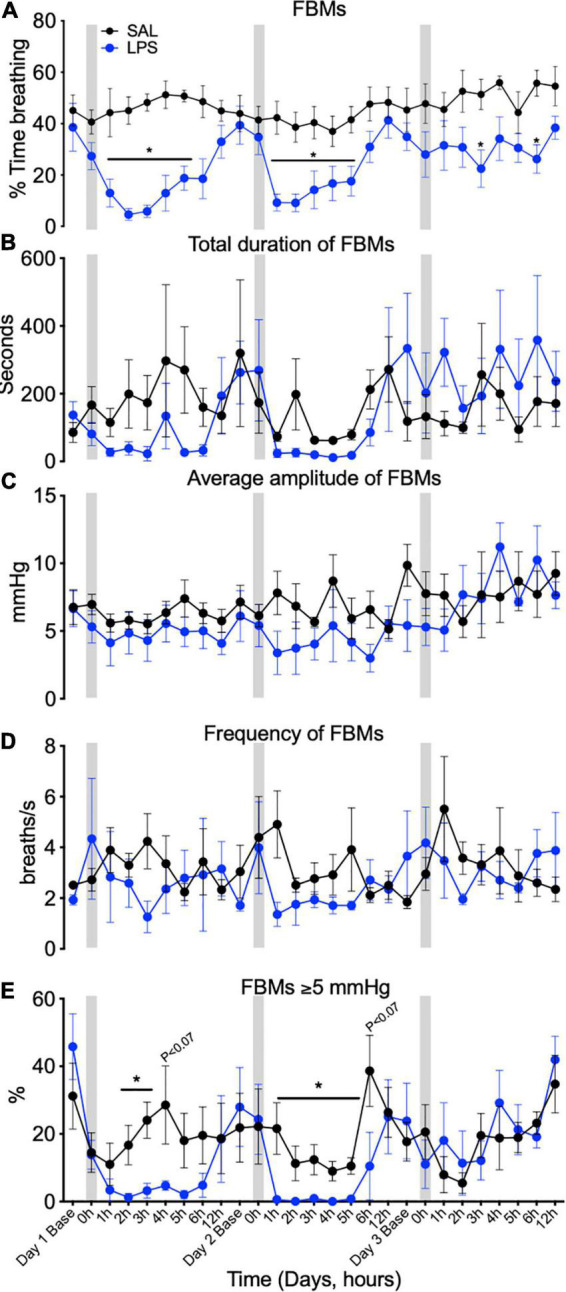
Fetal breathing movements (FBMs) measured across 3 days at baseline (1 h before SAL/LPS infusions), 0 h (the hour of SAL/LPS infusion), +1, 2, 3, 4, 5, 6, and 12 h epochs following saline (SAL) and LPS infusions. Percentage of time fetuses spent breathing **(A)**, total duration of FBMs **(B)**, average amplitude of FBMs (average depth of breaths during each 1-h epoch, **C**), frequency of FBMs (average number of breaths/second during an episode of FBMs over each 1 h epoch, **D**), and percentage of vigorous fetal breathing movements ≥ 5 mmHg **(E)** in SAL (control = black) and LPS-exposed (blue) fetuses. Gray bars indicate SAL or LPS infusion on each day after baseline sampling. Data are means ± SEM, *P < 0.05 vs. control.

In controls, FBMs occurred 40–60% of the time throughout the experimental period (Figure 2A). In LPS-exposed fetuses, FBMs were reduced compared to control on days 1 (by 89–74% between 1 and 5 h), 2 (by 79–57% between 1 and 5 h, *P* < 0.05), and 3 (by 57% and 54% at 3 and 6 h, respectively) after LPS infusions (*P*treat < 0.01, *P*treat × time < 0.00, Figure 2A). No differences between groups were observed for the duration, amplitude or frequency of FBMs (Figures 2B–D). In LPS-exposed fetuses the proportion of vigorous FBMs (≥5 mmHg) was reduced compared to control after the first and second LPS infusions (*P*treat < 0.11, *P*treat × time = 0.01, day 1, 2–3 h and day 2, 1–5 h, Figure 2E).

### PGE_2_ Levels in Plasma and Cerebrospinal Fluid

**FIGURE 3:**
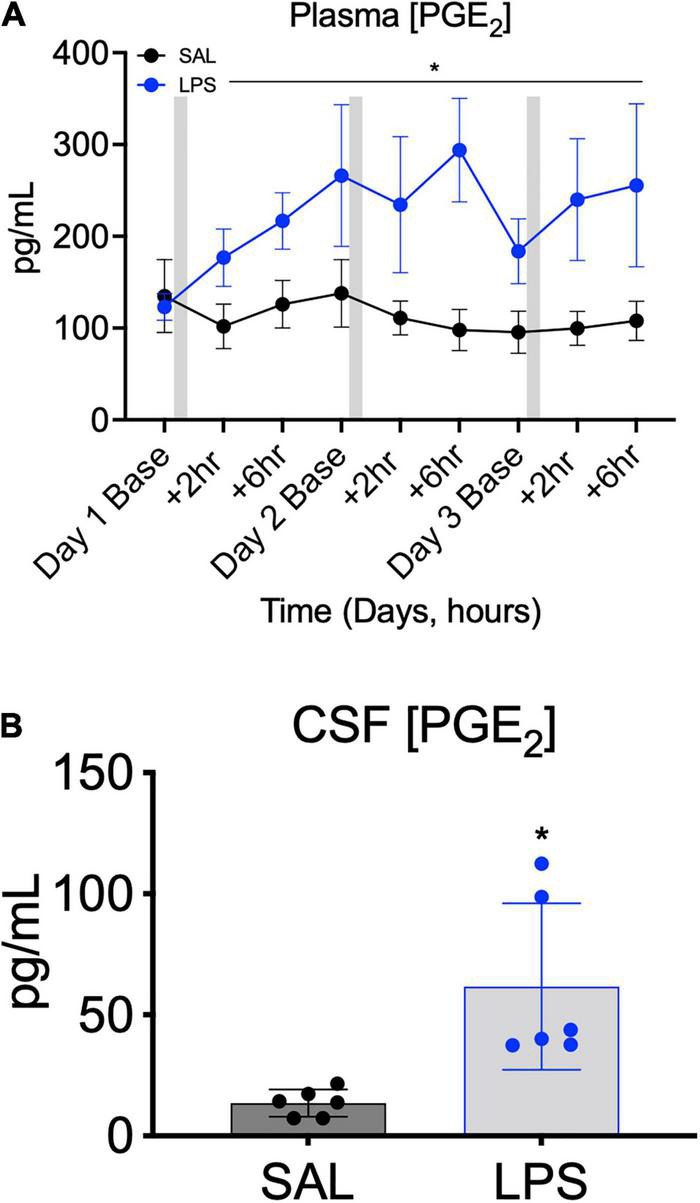
Plasma PGE_2_ concentrations measured across the 3 days of SAL/LPS infusions at baseline (before infusions), +2 and +6 h after infusions **(A)**. Gray bars indicate SAL or LPS infusion on each day after baseline sampling. Data are means ± SEM. Cerebrospinal fluid (CSF) PGE_2_ concentration in SAL (control = black, *n* = 6) and LPS-exposed (blue, *n* = 6) fetuses measured on experimental day 5 (4 days after starting infusions, **B**). Data are means ± SD, *P < 0.05 vs. control.

PGE_2_ concentration in plasma was higher in LPS-exposed lambs compared to controls from +2 h after the first LPS infusion until +6 h after the third LPS infusion (*P*treat = 0.04, *P*treat × time = 0.4, Figure 3A). On average, plasma PGE_2_ concentration was increased by 1.7-fold from 2 h after the first LPS infusion and remained elevated (by approximately 2.4–fold) throughout the 3-day experimental period (Figure 3A). CSF PGE_2_ concentration was 3-fold higher at post-mortem in LPS-exposed fetuses compared to controls (*P* < 0.05, Figure 3B).

### PGE_2_ Expression in Brainstem Respiratory Centers

**FIGURE 4:**
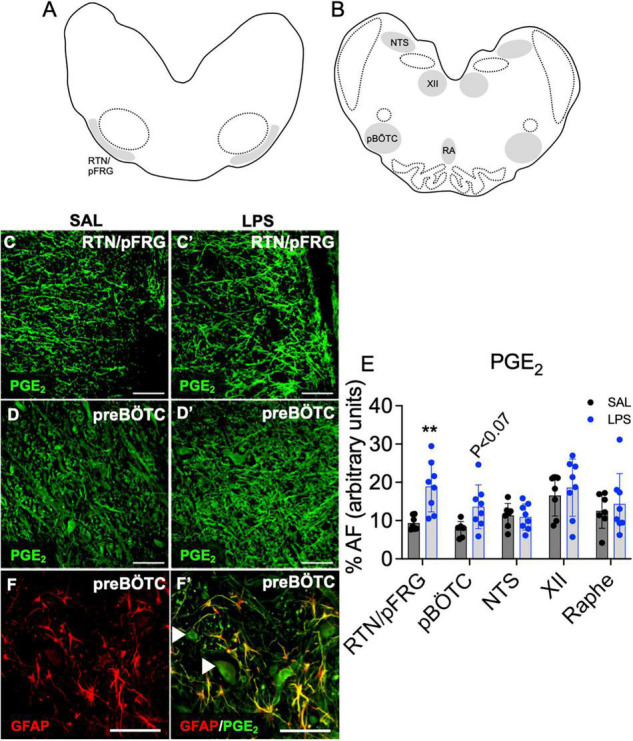
PGE_2_ expression in brainstem respiratory centers. Schematic diagram of brainstem respiratory centers analyzed: RTN/pFRG **(A)**, preBÖTC, NTS, XII, and raphe nucleus **(B)**. Representative photomicrographs of PGE_2_ (green) staining in the RTN/pFRG in SAL **(C,C’)** and LPS exposed fetuses **(D,D’)**. Area fraction of PGE_2_ staining in brainstem respiratory centers in SAL (control = black, *n* = 8) and LPS-exposed fetuses (blue, **E**, *n* = 8). Data are means ± SD, **P < 0.01 vs. control. Representative photomicrographs of GFAP (red) merged with PGE_2_ (green) showing co-localization of PGE_2_ to GFAP + astrocytes within the preBÖTC. White arrowheads indicate PGE_2_ positive neurons **(F,F’)**. Scale = 20 μm.

The % area coverage of PGE_2_ immunolabelling within the brainstem was significantly increased in the RTN/pFRG in LPS exposed fetuses compared to controls (*P* < 0.05, Figures 4A,C,C’,E). PGE_2_ expression within the preBÖTC was not significantly increased in LPS-exposed fetuses compared to controls (*P* < 0.07, Figures 4B,D,D’,E). PGE_2_ immunolabelling appears to be expressed in both neurons and glia. Double-immunolabeling showed co-localization of PGE_2_ with GFAP + astrocytes within the preBÖTC (Figures 4F,F’).

### Cyclooxygenase 2, Prostaglandin E Synthase, and Caspase 3 Gene Expression

**FIGURE 5:**
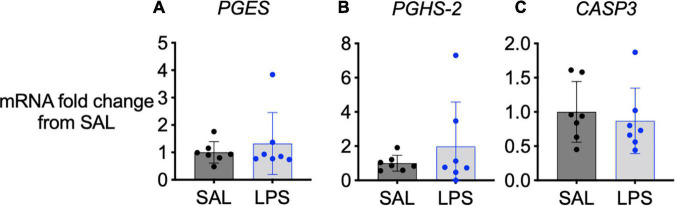
mRNA levels of *PGES* **(A)**, *PGHS-2* **(B)**, and *CASP3* **(C)** in the medulla of SAL (black, *n* = 7) and LPS-exposed fetuses (blue, *n* = 7). Data are means ± SD and expressed as fold change from the SAL (control) group.

No significant changes in COX-2 (*PGHS-2*), prostaglandin E synthase (*PGES*) or caspase 3 (*CASP3*) mRNA gene expression were observed in the medulla between groups (Figures 5A–C).

### Astrocytes and Microglia Within Brainstem Respiratory Centers

**FIGURE 6:**
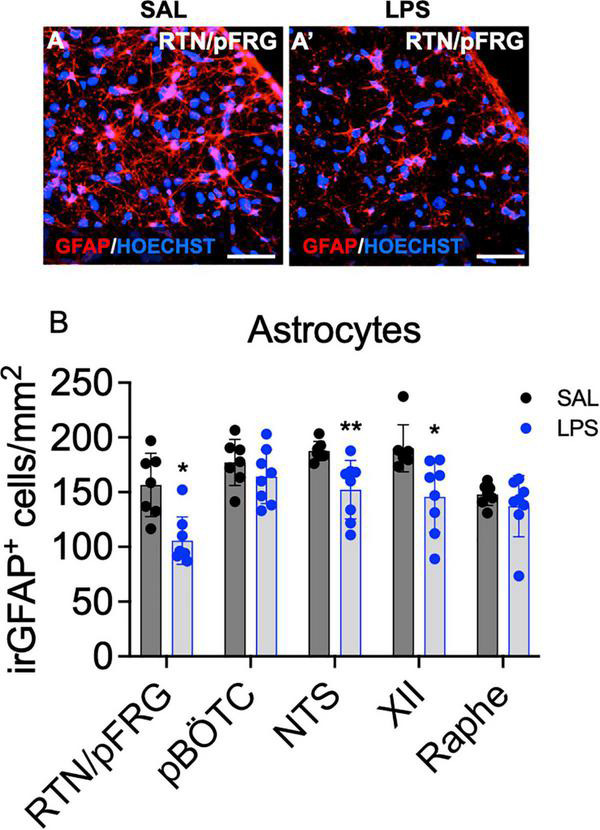
GFAP^+^ astrocytes within brainstem respiratory centers. Representative photomicrographs of GFAP + labeling (red) and the nuclear stain HOECHST (blue) in the RTN/pFRG of a control **(A)** and LPS exposed fetus **(A’)**. Scale = 20 μm. Numbers of GFAP + astrocytes in the brainstem respiratory centers in SAL (control = black, *n* = 8) and LPS-exposed fetuses (blue, **B**, *n* = 8). Data are mean ± SD, *P < 0.05 vs. control, **P < 0.01 vs. control.

The number of immunoreactive (ir) GFAP + astrocytes was reduced within the RTN/pFRG, XII nucleus and NTS in LPS-exposed fetuses compared to controls (*P* < 0.05, Figures 6A,B). The number of astrocytes within the preBÖTC and Raphe nuclei did not differ between groups (Figure 6B).

**FIGURE 7:**
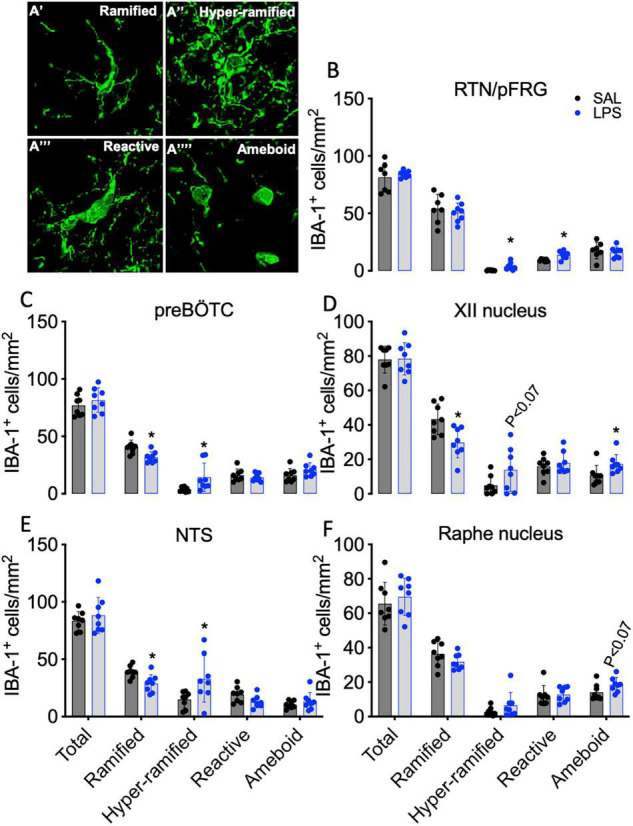
IBA-1^+^ microglia within brainstem respiratory centers. Representative images of microglial phenotypes assessed **(A)**. Total numbers of IBA-1 + microglia and numbers of ramified, hyper ramified, reactive, and ameboid IBA-1 + microglia in the RTN/pFRG **(B)**, preBÖTC **(C)**, XII **(D)**, NTS **(E)**, and raphe nucleus **(F)** in SAL (control = black, *n* = 8) and LPS-exposed fetuses (blue, *n* = 8). Data are means ± SD, *P < 0.05 vs. control.

Total IBA-1 + microglia, ramified (resting), hyper-ramified, reactive (stress-primed), and ameboid (phagocytic) IBA-1 + microglia were quantified based on their morphological phenotype (Figures 7A’–A””). The total number of microglia did not differ between groups for all brainstem regions analyzed (Figures 7B–F). The number of ramified microglia was reduced in the preBÖTC, XII, and NTS nuclei in LPS-exposed fetuses compared to controls (*P* < 0.05 for all, Figures 7C–E). The number of hyper-ramified microglia was increased in the RTN/pFRG, preBÖTC, and NTS following LPS-exposure (*P* < 0.05 vs. control, Figures 7B,C,E). In the XII nucleus, the number of hyper-ramified microglia was not significantly increased in LPS-exposed fetuses compared to controls (*P* < 0.07, Figure 7D). The number of reactive microglia was increased in the RTN/pFRG in LPS-exposed fetuses compared to controls (*P* < 0.05, Figure 7B). In LPS-exposed fetuses, numbers of amoeboid microglia were increased in the XII nucleus compared to controls (*P* < 0.05, Figure 7D). The number of amoeboid microglia in the raphe nucleus was not significantly increased in LPS-exposed fetuses compared to controls (*P* < 0.07, Figure 7F).

## Discussion

The present study demonstrates that progressive LPS-induced inflammation in late preterm fetal sheep decreases FBMs and increases PGE_2_ levels in the brainstem, CSF, and plasma. Further, progressive fetal inflammation resulted in hypoxia and hypercapnia, astrocyte loss and changes in microglial phenotypes that were characteristic of microglial activation in brainstem respiratory centers. Clinically, perinatal infection/inflammation is associated with reduced neonatal respiratory drive (Siljehav et al., 2015). However, the effects of antenatal inflammation on fetal breathing are not well known. Previous research has demonstrated that the inflammatory mediator PGE_2_ modulates breathing when administered systemically, and at the level of the brainstem (Wlodek et al., 1998; Hofstetter et al., 2007; Siljehav et al., 2012; Siljehav et al., 2014; Siljehav et al., 2015). In this study, we aimed to determine whether progressive fetal inflammation, caused by repeated exposure to increased doses of LPS, inhibits FBMs and whether changes in breathing are associated with increased PGE_2_ levels and brainstem inflammation.

Previous studies have shown that inflammatory mediators, particularly PGE_2_, can inhibit breathing in fetal sheep and alter hypoxic and hypercapnic responses in neonatal mice (Wlodek et al., 1998; Alvaro et al., 2004; Hofstetter et al., 2007; Siljehav et al., 2014; Siljehav et al., 2015). Mechanistic insights into how PGE_2_ affects breathing come from acute studies, whereby PGE_2_ has been directly infused into fetal sheep or applied to brainstem slices in rodents. While there is strong evidence that PGE_2_ is negatively associated with breathing, these acute animal studies do not replicate the conditions of chronic progressive inflammation, which occurs in a large proportion of preterm infants (Moss et al., 2005; Sweeney et al., 2017; Oh et al., 2019). We found that progressive inflammation in late preterm fetal sheep inhibits FBMs across the 3-day exposure period. Our data are consistent with outcomes from clinical studies in which the absence of FBMs is an indicator for premature rupture of membranes and intrauterine inflammation (Vintzileos et al., 1985; Jaschevatzky et al., 1986; Besinger et al., 1987).

In this study we observed increases in PGE_2_ expression within the RTN/pFRG and a trend toward an increase in PGE_2_ in the preBÖTC (*P* < 0.07). Previous studies have shown that PGE_2_ has differential effects on these brainstem respiratory centers. The RTN/pFRG is responsible for rhythmic generation of active expiration and central chemosensitivity (CO_2_ and pH), whereas the preBÖTC is responsible for rhythmic generation of inspiratory drive. PGE_2_ application to brainstem slices containing the RTN/pFRG increases astrocytic and neuronal activity, however in contrast, inhibits preBÖTC neurons (Forsberg et al., 2016). The different actions of PGE_2_ on these respiratory centers may be due to different eicosanoid prostaglandin receptor 3 (EP3R) subtypes which can be coupled to stimulatory or inhibitory G proteins (Namba et al., 1993). These receptor subtypes have not been extensively explored in the brainstem respiratory centers. However, it is known that EP3R is critical for mediating respiratory changes in response to PGE_2_. Indeed, neonatal mice lacking the EP3R exhibit fewer apnoeas and neuronal activity within the preBÖTC is preserved following PGE_2_ exposure (Siljehav et al., 2014). Further, we observed increases in PGE_2_ levels within the CSF and plasma in fetal sheep exposed to LPS. Our data agree with previous research in human preterm infants exposed to perinatal infection/inflammation, which have correlated high CSF PGE_2_ levels with breathing difficulties (apnoeas and hypoxic events) (Siljehav et al., 2015). Overall, these data strongly suggest that PGE_2_ is a key modulator of fetal breathing and potentially reduces a newborn infant’s stimulus to breathe after birth, thereby increasing their requirement for respiratory support. Future work should aim to investigate whether PGE_2_ blockade restores fetal and neonatal breathing during perinatal inflammation.

In this study, we observed significant reductions in SaO_2_ in LPS-exposed fetal sheep over the 3-day experimental period and we have previously reported increased production of pro-inflammatory cytokines, including IL-1β (Kelly et al., 2021). Hypoxia and IL-1ß-induced inflammation rapidly increases microsomal prostaglandin E-synthase 1 (mPGES-1) expression within the rodent brainstem (Hofstetter et al., 2007). mPGEs-1 is the enzyme which converts prostaglandin H2 to PGE_2_, highlighting that PGE_2_ may be upregulated by both inflammation and hypoxia. Interestingly, we did not observe significant increases in *PGHS-2* or *PGES* gene expression in the medulla of LPS-exposed fetal sheep, despite their hypoxic and inflammatory states. Nonetheless, the increased levels of PGE_2_ within the brainstem, CSF and plasma indicate that the activity of PGE_2_ enzymes are increased. Notably, suppression of FBMs was attenuated after the third LPS infusion at which point plasma PGE_2_ levels were still elevated. This raises the possibility that other biochemical factors may be involved in regulating the inflammation-induced suppression of FBM. In line with reduced suppression of FBM after the third LPS infusion we observed a reduction in the magnitude of hypoxia, as shown by a higher oxygen saturation in LPS-exposed fetuses compared to the first and second LPS infusions. It is well known that hypoxia inhibits fetal breathing (Koos et al., 1987; Herlenius, 2011). In addition to upregulating PGE_2_ levels, hypoxia also leads to adenosine production (Koos et al., 2002). Adenosine inhibits FBMs by inhibiting the activity of expiratory and inspiratory neurons within the brainstem (Herlenius et al., 1997; Herlenius and Lagercrantz, 1999; Koos et al., 2002; Falquetto et al., 2018), whereas caffeine, an adenosine receptor antagonist, is routinely given as a respiratory stimulant to preterm infants. Thus, a reduction in hypoxemic adenosine release may have contributed to attenuated suppression of FBM after the third LPS injection. We have not exclusively explored hypoxic pathways or adenosine levels in this study, but it should be noted that previous work has demonstrated that inflammation can induce hypoxia and adenosine production (Cronstein, 1994; Galinsky et al., 2018a) and both hypoxia and adenosine can inhibit fetal breathing (Bissonnette and Hohimer, 1987; Koos et al., 1994; Koos et al., 2002).

Lipopolysaccharide infusions were associated with hypercapnia and acidosis. The LPS-induced hypoxemia, hypercapnia, and acidosis are most likely mediated by inflammation-induced vasoconstriction of the placental vascular bed. This may have contributed to the systemic and brainstem inflammatory responses *via* hypoxia-induced inflammation (Dalitz et al., 2003). Moreover, we observed a reduction in the magnitude of LPS-induced hypoxemia, hypercapnia and acidosis, particularly after the third LPS infusion. This is consistent with previous studies from us and others that have shown tolerance to subsequent LPS doses (Feng et al., 2009; Feng et al., 2010; Lear et al., 2014; Lear et al., 2015; Galinsky et al., 2020a; Kelly et al., 2021). The immune tolerance is in part mediated by reprogramming of the innate immune system as shown by studies in human and sheep monocytes, whereby repeated LPS exposure is associated with decreased cytokine production and downregulation of the LPS receptor CD14 (Mengozzi et al., 1993; Kramer et al., 2009).

While both hypercapnia and acidosis can stimulate FBMs, this effect was not observed in the LPS-exposed fetal sheep. This may be due to the strong inhibitory effect of hypoxia which is known to override the stimulatory effect of hypercapnia and academia (Koos et al., 1994). Further, the RTN/pFRG and raphe nucleus are the main chemosensitive regions in the brainstem which respond to changes in CO_2_ and pH levels. Astrocytes within the RTN/pFRG are pH-sensitive and release ATP to stimulate RTN/pFRG neurons and expiratory drive (Gourine et al., 2010; Mulkey and Wenker, 2011; Forsberg et al., 2017). In this study, we observed a significant reduction in the number of ir-astrocytes within the RTN/pFRG. The reduction in astrocytes within this respiratory region may have contributed to the impaired adaptive responses to the inflammation-induced increase in CO_2_ and pH levels, however, this remains to be studied.

Indeed, in the brainstem astrocytes play an important role in modulating neuronal activity within the respiratory centers. In this study, we have shown that progressive fetal inflammation caused a significant reduction in numbers of ir-astrocytes within the RTN/pFRG, NTS, and XII nuclei. Astrocytes are critical for central chemosensory functions. Indeed, RTN/pFRG astrocytes have been linked to respiratory drive modulation (Gourine et al., 2010; Mulkey and Wenker, 2011; Forsberg et al., 2017). In the NTS, astrocytes facilitate the processing and coordination of peripheral chemosensory information (O_2_, CO_2_, and pH) (Smith et al., 2013; MacDonald and Ellacott, 2020). The XII nucleus, in addition to the ventral respiratory group (including the preBötzinger nucleus), is integral in the control of upper airway patency to regulate the passage of air into and out of the lungs (Smith et al., 2013). The reduction in the number of ir-astrocytes within these brainstem regions likely represents the acute phase of injury during neuroinflammation. For example, we and others have previously reported reduced numbers of astrocytes within 48–96 h after LPS-induced fetal inflammation in sheep, hypoxia-ischemia in neonatal piglets and mechanical ventilation in preterm lambs (Martin et al., 1997; Nott et al., 2020; Kelly et al., 2021). We also showed that PGE_2_ was co-localized with GFAP^+^ astrocytes. These data are consistent with previous work that has shown astrocytes are a source for PGE_2_ in the brainstem (Forsberg et al., 2017). Further work is required to understand the effects of astrocyte loss and astrocyte induced PGE_2_ release within these respiratory centers.

We observed morphological changes in microglia within the medullary respiratory centers after LPS-exposure that were characteristic of microglial activation. Aside from their immunological functions, microglia are important for axonal development and synaptic pruning and plasticity (Nayak et al., 2014). In this study, LPS exposed fetuses had a reduction in the numbers of ramified microglia in the preBÖTC, NTS, and XII nuclei. By contrast, numbers of hyper-ramified, reactive, and ameboid microglia were increased within the preBÖTC, NTS, RTN/pFRG, and XII nuclei. A change in microglial morphology represents a key characteristic of the microglial response to inflammatory and/or stressful stimuli. The hyper-ramified and reactive phenotypes are known as “stress-primed” microglia, whereas the reactive/ameboid microglia are known to have phagocytic functions (Stence et al., 2001). These data demonstrate that LPS-exposure is associated with microglial responses within the brainstem respiratory centers that are characteristic of microglial activation and inflammation. Indeed, PGE_2_ induced activation of microglia has been associated with impairments in microglial homeostasis, characterized by disturbances in microglial chemotaxis, cytotoxic inflammation, inhibited production of trophic factors, and synaptic injury (Johansson et al., 2015). Collectively, these data suggest that microglial activation may play a vital role in promoting disturbances in brainstem function that contribute to reduced respiratory drive in the fetus and neonate and that PGE_2_ inhibition could improve microglial function. However, it is important to acknowledge that histological assessments of brainstem inflammation and injury were undertaken approximately 32 h after observing LPS-induced disturbances in FBMs. Thus, future investigations should focus on whether disturbances in brainstem pathology can be identified at the time of FBM inhibition.

## Conclusion

In conclusion, LPS-induced inflammation inhibited FBMs, increased PGE_2_ levels within the brainstem, CSF and plasma, and lead to astrocyte loss and microglial activation within the brainstem respiratory centers. These data demonstrate an important link between inflammation-induced increases in brainstem PGE_2_ levels and inhibition of fetal breathing and support previous studies in human preterm infants exposed to perinatal infection/inflammation that demonstrated an association between increased circulating and cerebrospinal fluid prostaglandins and inhibition of breathing. Based on these data, further translational studies are needed to evaluate whether antenatal and or postnatal PGE_2_ inhibition is an effective intervention to reduce brainstem inflammation and injury and restore FBMs, improve neonatal respiratory drive and reduce the need for inadvertently injurious respiratory support at birth.

## Data Availability Statement

The raw data supporting the conclusions of this article will be made available by the authors, without undue reservation.

## Ethics Statement

The animal study was reviewed and approved by Hudson Institute of Medical Research Animal Ethics committee.

## Author Contributions

VS contributed to the conceptualization, methodology, data analysis, drafting, and revision of the manuscript. JA, VZ, SK, EM-S, IN, AM, YP, SH, and EH contributed to technical and project support, data analysis, and critical manuscript revision. RG and GP were responsible for conceptualization, methodology, data analysis, technical and project support, funding acquisition, manuscript revision, and supervised all aspect of the project. All authors contributed to the article and approved the submitted version.

## Conflict of Interest

The authors declare that the research was conducted in the absence of any commercial or financial relationships that could be construed as a potential conflict of interest.

## Publisher’s Note

All claims expressed in this article are solely those of the authors and do not necessarily represent those of their affiliated organizations, or those of the publisher, the editors and the reviewers. Any product that may be evaluated in this article, or claim that may be made by its manufacturer, is not guaranteed or endorsed by the publisher.

## Footnotes

1
https://msu.edu/b̃rains/brains/sheep/index.html
